# Overview of Legal and Policy Framework Approaches for Plastic Bag Waste Management in African Countries

**DOI:** 10.1155/2020/8892773

**Published:** 2020-10-31

**Authors:** Brian Nyathi, Chamunorwa Aloius Togo

**Affiliations:** Department of Environmental Management, University of South Africa, Private Bag X6, Florida 1710, South Africa

## Abstract

Plastic bag waste is a major challenge in several African countries. As a way of reducing the detrimental effects posed by plastic bags, governments have adopted various approaches for plastic bag waste management that include levies, bans and or the combination of the two. Despite the adoption of anti-plastic bag policies in several African countries, there has been limited investigation regarding their effectiveness. Thus, the present paper reviews the effectiveness of legal and policy framework approaches for plastic bag waste management in African countries. This systematic review covers legal and policy framework approaches for plastic bag waste management in African countries since 2004 with a view to find their effectiveness. Data sources included peer-reviewed journal articles, websites, books, reports, and dissertations. The databases from which literature was retrieved included Elsevier, Taylor, Springer, institutional repository, and Google Scholar. The current paper argues that poorly enforced plastic bag legislation, resistance from stakeholders, and limited effective substitutes are major factors hindering effective plastic bag waste management in Africa. A six-step model developed by Patton and Sawicki assisted in evaluating plastic bag policies in varied African nations. The study concluded that the key to effective legislation is hinged on consistent enforcement and educating the public to attain environmental buy-in. African countries should adopt and implement the Patton and Sawicki six-step rationalist model to achieve effective plastic bag legislation. African governments should enlighten consumers about other alternatives that are more specific to African countries, especially bags made of raffia and leaves. The findings are limited in that there may be other relevant articles (beyond published articles) about policy and legal approaches for plastic bag waste management, which are not available in the public domain. Therefore, data reviewed may not be exhaustible.

## 1. Introduction

Plastic bag waste is linked to environmental and socioeconomic problems [1, 2]. They are responsible for the clogging of water drains. Blocked water reticulation systems cause water bursts that lead to excessive loss of water [3–5]. This has economic repercussions as local authorities have to repair or acquire new water pipelines. Furthermore, plastic bag waste has been linked to the spread of disease; they create standing water for mosquitoes [5–7]. The light nature of plastic bags makes them windblown, causing loss of aesthetic value in the environment [8–10].

Plastic bag pollution causes negative environmental effects in several African countries for example, Botswana, Uganda, Kenya, and Zimbabwe [11–14]. They negatively affect the aesthetic quality of the environment, block water channels, and are responsible for the deaths of some animals. Animals and livestock often mistake plastic bags for food and often die after ingesting discarded plastic bags. In Gondar city, Ethiopia, plastic bags were found in stomachs of cows in abattoirs [15]. Plastic bag waste in landfills leaches into water sources causing soil pollution. Plastic bag waste promotes breeding ground for mosquitoes that cause malaria [10]. Based on literature review, plastic bag pollution has detrimental effects on the environment.

In 2015, plastic bags and other plastic packaging waste accumulated and clogged drains during a heavy downpour in Ghana Accra. This scenario resulted in flooding in which at least 150 people lost their lives and infrastructure was damaged [16]. Improperly disposed plastic bag waste promotes the spread of diseases. Diseases such as malaria and Zika virus can easily spread [17].

Plastic bag waste is made of toxic chemicals [18]. The decomposition of plastics in landfills cause environmental pollution [19]. Incinerated plastic bag litter releases dangerous pollutants such as furans and dioxins that have adverse effects on life [1, 20]. Some plastic bags contain toxic chemical additives, including persistent organic pollutants (POPs), which have been linked to health issues such as impairment of the nervous system, leukemia, skin diseases, cancer, eye irritation, vision failure, and developmental and reproductive diseases [21]. Microplastics can accumulate in food chains through water supplies and agricultural soils. As a result, this leads to health issues (oxidative stress, necrosis, and inflammation) linked to cardiovascular disease and cancer, if ingested by humans [1, 20]. Considering the previously mentioned observations, it can be concluded that improperly discarded plastic bag waste causes health problems, hence the need to regulate them.

**Figure 1:**
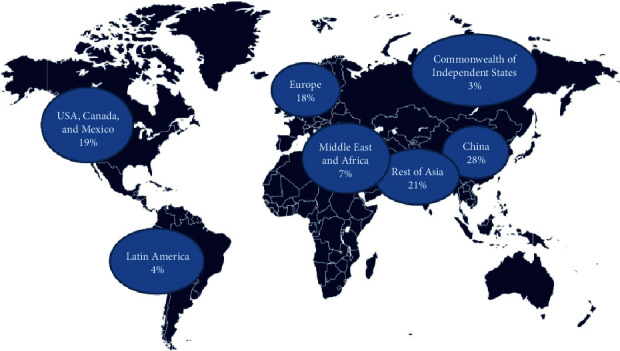
Global distribution of plastics production [21].

**Figure 2:**
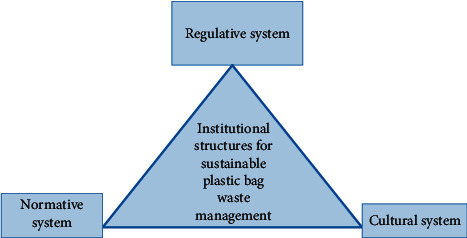
Institutional structures for effective plastic bag sustainable waste management [22–25].

Fears have been raised about the increasing volume of plastic production worldwide (Figure 1), and governments have adopted regulatory, normative and cultural systems to control the problem (Figure 2). Africa and Middle East share approximately 7% distribution of plastics production. Globally, between 1950 and 2015, there was a significant increase in manufactured plastics from 2 million tonnes to 380 million tonnes [1]. The volume of plastics in oceans is expected to increase significantly by 2025 [21]. This is of environmental concern as plastic packaging materials are a threat to aquatic life. The findings reveal that Asia and Europe are the leading continents in plastic production; however, plastic remains an environmental issue in Africa because of the environmental hazards they cause.

Governments have enacted laws to reduce the detrimental effects of plastic bag litter. These measures include levies and bans and the hybrid of the two [26, 27]. In Africa, several countries have enacted plastic bag ban legislation; however, plastic bag pollution is still a challenge [5, 8, 9, 12, 28]. The previously mentioned disposition has spurred the authors to provide a review of legal and policy framework approaches for plastic bag waste management in African countries.

Several studies [27, 29, 30] are based on legal and policy approaches for plastic bag waste management in developed countries. The current review paper takes a unique approach by examining literature on legal and policy approaches for plastic bag waste management in African countries. To achieve this objective, the researcher sought to answer the following research questions: how effective are legal and policy framework approaches for plastic bag waste management in African countries? The main research question was answered through the following subquestions.

- Which African countries have enacted plastic bag legislation?
- Are enforcement and monitoring systems effective?
- Is the public accepting or resisting plastic bag legislation?

While the current paper review offers novel insights about policy and legal approaches for plastic bag waste management in Africa, it also has limitations.

## 2. Methodology

### 2.1. Protocol

The protocol included eligibility criteria, information sources, study selection, data collection process, data items, risk of bias in individual studies, and data analysis. This systematic review was informed by Cochrane handbook guidelines and was reported in line with PRISMA requirements [31].

### 2.2. Eligibility Criteria

Relevant reports, books, websites, and peer-reviewed journal articles about policy and legal approaches for plastic bag waste management in Africa (from 2004 to date), policy analysis, and lessons from successful global policies were independently reviewed at the title and abstract level by two authors. Plastic bag legislation in Africa dates back from 2004 (for example Rwanda and South Africa) up to date. Any differences were resolved by discussion until consensus was reached. A list of African countries that have enacted plastic bag laws and policies was collected from several researchers [5–8]. Information sources were done by keyword searches such as plastic bag legislation in Africa, using Google.

### 2.3. Data Items

Data sources included 57 relevant peer-reviewed reports, websites, books, and peer-reviewed journal articles about policy and legal approaches for plastic bag waste management in Africa, policy analysis, and lessons from successful global policies. The databases from which literature was retrieved included the following: Elsevier, Taylor, Springer, Google Scholar, institutional repository, reports, and books. Information was collected by key word searches derived from expertise in the subject field (for example, plastic regulation in Africa) using Google. The two authors independently searched for data sources. Disagreements about data selection were resolved through discussion between the two authors [31].

Data about African countries that have enacted legislation to control the use of plastic bags was presented in tabulated form. Data was grouped according to outcome to evaluate effectiveness of policy and legal approaches for plastic bag waste management in African countries. Data analysis included a process for synthesising, analysing, and presenting descriptive summaries. Results were grouped based on African countries that have enacted plastic bag legislation and effectiveness of plastic bag policies (enforcement and monitoring systems and public acceptance or resistance of plastic bag legislation).

## 3. Theoretical Framework

Theories help to understand, explain, and make predictions about a given subject matter [32, 33]. The institutional theory helps guide and understand that the human behaviour can be controlled by the regulatory pillar. The regulatory pillar is also interconnected to normative and cultural pillars. All these pillars influence the effective implementation of any plastic bag legislation [34, 35].

### 3.1. Institutional Theory

According to the institutional theory, institutional structures control humans (Figure 2), namely, regulatory, normative, and cultural systems [35, 36]. These social structures shape behaviour and restrict certain human actions [36]. Although regulatory, normative, and cultural systems are regarded as important components of institutions, it is important to consider individual's behaviour and material resources as significant factors that can influence sustainable plastic bag waste management [22, 23].

The regulative pillar constrains and regularises human behaviour [25]. Government and local authorities govern and enforce laws on the public. However, legislation must be interpreted, conflicts resolved, and surveillance measures put in place to achieve intended objectives [23, 37].

The normative pillar underlines the importance of values and norms in shaping human behaviour [22, 35, 36]. Normative pillar consists of both values and norms. Values are conceptions of the desired while norms specify how people are supposed to behave [37, 38].

The cultural cognitive element gives emphasis to cultural legitimacy based on a common reference [38]. Although norms, values, rules, and cultural beliefs are key ingredients of institutions, the theory must also consider material resources and individual's behaviour [22–24].

The researcher examined all legal and policy approaches for plastic bag waste management based on the regulative pillar and determined the level of private and public participation guided by the influence of cultural and normative systems. As suggested by [23], the researcher took into consideration material resources individual's behaviour as determinants of effective plastic bag legislation. Institutions can be weakened by exogenous factors such as economic crises [39]. Many researchers [26, 40, 41] have established that sustainable waste management is dependent and deeply rooted in institutional structures (regulative, normative, and cultural systems) in most African countries such as Tanzania, Uganda, and South Africa. African governments have enacted laws to govern consumption of plastic bags.

## 4. Policy Analysis

Policy analysis is a process of identifying and evaluating policies meant to reduce or manage socioeconomic and physical problems [42]. Policy analysis is vital in identifying and evaluating plastic bag policies. A six-step model developed by [42] was adopted (Table 1) in determining the effectiveness of plastic bag policies in several African countries. The model can help develop effective policies that can help manage plastic bag waste problems in Africa.

A six-step model developed by [42] was adopted to determine the effectiveness of plastic bag policies in several African countries. Several researchers have used political acceptability, compliance, and monitoring systems to determine the effectiveness of plastic bag management policies [5, 6, 9].

Thus, the researchers made use of factors such as political acceptability, compliance, and monitoring systems to determine the success and failure of legislation in countries such as Zimbabwe, South Africa, Botswana, and Rwanda.

## 5. Results

### 5.1. Plastic Bag Legislation in Africa

The African continent has the highest number of countries that has enacted legislation to control the use of plastic bags. Some of these countries include Mali, Kenya, Zimbabwe, Guinea-Bissau, Ethiopia, Eritrea, Tanzania, Chad, Cameroon, Mauritania, Morocco, Niger, Somalia, Rwanda, Tunisia, Mozambique, Botswana, and South Africa (Table 2) [5, 8, 14, 43]. Legislation enforcement remains a challenge to many African countries mainly because of stakeholder resistance and inconsistent/weak enforcement [5, 6, 16].

Poorly enforced and outdated waste management legislation has been noted as one factor hindering sustainable plastic bag waste management in Africa [44]. In Zimbabwe, legislation pertinent to solid waste management was previously administered by more than four government ministries. Following the enactment of the Environment Management Act (EMA) (Chapter 20 : 27) into law in 2002, environmental legislation such as Natural Resources Act (Chapter 20 : 13) and Natural Resources Act (Chapter 20 : 13) were replaced to bring about harmonization in environmental legislation.

### 5.2. Effectiveness of Plastic Bag Policies

Studies in several African countries such as Uganda, South Africa, Kenya, and Zimbabwe have enacted legislation to control the use of plastic bags [45–47]. Plastic bag ban legislation in some countries have been effective, while others have encountered limited or no noticeable changes [3, 48].

#### 5.2.1. Enforcement and Monitoring Systems

In Africa, Rwanda carries the torch in plastic bag waste management. The country has enacted strict laws prohibiting the use of single use plastic bags to the extent that travellers from abroad are not allowed to enter the country with plastic bags [49]. The Rwanda reinstated plastic bag ban in 2008 and the legislation is effectively enforced to date [9]. The legislation came into place because of plastic bag pollution and its impacts on the agricultural sector [14]. However, alternatives at that time did not exist and environmentally friendly bags were to be imported [50]. Other successful cases have been observed in Eritrea, where plastic bags have been replaced by cotton bags, and in Senegal, where recycling system has been implemented for the recovery of plastic products [5, 6, 9].

Burkina Faso and Madagascar enacted a ban on the use of thin plastic bags in 2014 and 2015, respectively [5, 51]. Problems that have hindered the effective legislation enforcement include stakeholder resistance, poor enforcement, and lack of substitutes [26]. Weak or poor plastic bag ban enforcement has been detected in DRC, Cote d'Ivoire, Ethiopia, Mali, Malawi, Niger, Morocco, and Tanzania [5, 6, 8, 9]. Inconsistent enforcement is still a challenge in many African counters, hence the need for a consistent enforcement monitoring system.

#### 5.2.2. Public Resistance of Plastic Bag Legislation

In 2017, Kenya reinstated a plastic bag ban. The ban came after the global call of managing plastic bag litter, which is blamed for disastrous effects on marine life and the environment in general [13]. However, the ban of plastic bags has been condemned by other stakeholders in waste management. Kenya Association of Manufacturers (KAM) challenged the plastic bag ban legislation arguing that if upheld, it would lead to immense losses in the affected companies. The court, however, dismissed the suit holding that if KAM's orders were granted, the plastic bags would bring about negative environmental problems [52].

For legislation to be effective, stakeholders must be adequately involved as this gives them a sense of belonging and to be committed to the successful implementation of plastic bag legislation [53, 54]. Plastic bag ban resistance has been observed in other counties like Zimbabwe, Benin, DRC, Ethiopia, Ghana, Mali, and Uganda [5, 8, 9]. Effective legislation is hinged on consistent enforcement and educating the public to attain environmental buy-in [6]. Thus, governments and local authorities should do extensive public awareness programmes to enlighten consumers about the detrimental effects of plastics and the need to use substitutes such as cotton bags and bags made from leaves or raffia as they are environmentally friendly.

Botswana adopted a plastic bag ban legislation and levy to reduce the detrimental effects of plastic bags [55]. The introduction of the plastic levy in Botswana caused a decline in the consumption of plastic bag [48, 55]. The plastic bag ban was challenged by manufacturers who argued that they were not adequately consulted before the ban was enacted and that there was no justification concerning plastic bag substitutes [56]. The ban had limited success and was reinforced in 2017.

South Africa introduced a plastic bag levy of 46 Rand cents in 2003, which was afterwards reduced to 32 Rand cents, with the retailers and the public paying public 15 and 17 Rand cents, respectively. Following the reduction of the plastic bag levy, plastic bag consumption rose again [28, 57]. The country then enacted a ban on single-use plastic bags in 2004 [58]. The ban came because of the negative impacts associated with plastics on the environment [12]. The use of plastic bags dropped slightly, but then increased again because of law enforcement problems [12, 57].

Zimbabwe enacted a ban on all single-use plastic bags in a drive to reduce pollution [59]. Despite the growing unpopularity of plastic bags with Zimbabwean environmental groups supporting total ban, Mangizvo [57] is of the view that plastic regulation is better than total phasing out of thin plastic bags. Furthermore, the researcher called for adequate public consultation and extensive media coverage before the law is put in place and supports the idea of banning single-use plastic bags as they are difficult to recycle. However, legislation of plastic bag waste tends to be ineffective and inefficient if there is no strict enforcement and poor change management strategy. If government departments and local authorities are reluctant to prosecute people who violate provisions of the legislation, plastic bag waste management becomes a challenge.

## 6. Lessons from Successful Global Policies

Studies in several countries, such as England, Ireland, Netherlands, China, Philippine, and Australia, have shown that that plastic bag fee is effective in controlling plastic bag use [5, 6, 8, 60]. China introduced plastic bag fee of 0.20–0.50 CNY in 2008. He [61] observed a 64% decline in the consumption of new plastic bags by consumers when shopping. England introduced a plastic bag fee of 5 pence in 2015 on major businesses. Poortinga et al. [27] conducted a study in an independent supermarket and revealed a 36% decrease in plastic bag consumption following the introduction of the plastic bag fee. Portugal introduced a plastic bag tax of 0.10 Euro on plastic bags in 2015. Martinho et al. [29] found out that there was a great reduction (74%) in plastic bag use and an increase (61%) of reusable plastic bags in one of the supermarkets following introduction of the tax. Wales introduced a single-use plastic bag fee of 5 pence (0.07 USD) in 2011. The consumption of plastic bags fell by 70% in a variety of retail shops following the introduction of the plastic bag fee. The number of people who used their own bags improved by 21% from 61% [30].

Global cases from other countries who have implemented plastic bag fee have shown a significant reduction in plastic bag consumption [8, 60]. The combined effect of plastic bag tax and ban, strict enforcement, and educational awareness campaigns can help realise effective plastic bag policies [5, 6, 26].

## 7. Conclusion

Several African countries have enacted legislation banning use of lightweight plastic bags to reduce the detrimental effects of plastic bag litter. However, there is complexity around the application of legal or policy framework in Africa. Identified legislation enforcement impediments included poor/limited enforcement, legislation resistance, and lack of substitutes. All these factors have hindered effective and sustainable plastic bag waste management. The findings are limited in that there may be other relevant articles (beyond published articles) about policy and legal approaches for plastic bag waste management, which are not available in the public domain. As a result, data reviewed may not be exhaustible.

## 8. Summary Measures

The current paper has established that poorly enforced plastic bag legislation, resistance from stakeholders, and limited effective substitutes are major factors hindering effective plastic bag waste management in Africa.

A six-step model developed by [42] should be adopted and implemented by African countries as it is vital in determining the effectiveness of plastic bag policies. The model is fundamental in the development of policies that are effective and sustainable for African countries. Plastic bag policies should be based on tested approaches and communicated and understood by members of the public to ensure transparency. A mechanism for stakeholder involvement should be established before law enactment. Sound legal frameworks contribute to effective law enforcement.

Ministries involved in small and medium enterprises should encourage private business to engage in the manufacturing of environmental friendly bags such as cotton bags and bags made from raffia and leaves. Universities and colleges should establish programmes that train small business about business management and the manufacture of bags made from raffia and leaves (these are readily available; hydrocarbon-free and biodegradable).

Governments need to move away from conformance and compliance to conscious decision making based on understanding people's behavioural trends and their value in keeping the environment clean. Legislation must be interpreted, conflicts resolved, and surveillance measures put in place to attaining environmental buy-in and intended objectives. In that way, people can hold each other accountable.

Law enforcers should do routine monitoring to educate users and illegal traders about the negative impacts of plastic bags. Other strategies include the following:

- Conducting more foot patrols
- Developing strong connections and continually improve social networks into communities where there are no bonds
- Sharing policies, information, investigative results, and other important information about legislation
- Developing posters for antilittering and illegal dumping

The public should bring their own bags when shopping and actively partake in clean-up campaigns.

## Figures and Tables

**Table 1:** Six-step rationalist (adapted from [42]).

Step	Description
Step 1: identify the problem	This entails problem identification and description. Many African governments have enacted plastic bag policies/legislation after observing their detrimental effects in the environment (Jambeck et al., 2018). This step is vital and forms a foundation for any effective and efficient plastic bag policy
Step 2: establish evaluation criteria	Factors such as politically acceptance, public compliance, public awareness, policy enforcement, reduction or consumption of plastic bag, and legality are considered to evaluate how effective is a plastic bag policy [5, 8, 11, 26]
Step 3: identify alternative policies	Several policies should be identified and combined to ensure their effectiveness and efficiency. Data collected from different governmental documents and journals showed that governments employ different plastic bag policies with varied results. Case studies from countries such as Rwanda, Zimbabwe, South Africa, and Kenya have helped to establish key factors determining failure or success of plastic bag policies [5, 8, 9, 11]
Step 4: evaluate alternative policies	Alternative policies are evaluated based on political acceptance, public compliance, public awareness, policy enforcement, reduction or consumption of plastic bag, and legality to ensure their effectiveness [5, 9]
Step 5: indicate the most effective plastic bag policies	After the evaluation of alternatives, findings can be used to determine the most effective policies [5, 6, 8]
Step 6: monitoring the implemented policy	Policy analysis continues after policy implementation. After policy implementation, a monitoring system has to be developed to monitor the effectiveness of plastic bag policy. Policy analysis helps establish whether the policy is being correctly implemented and helps determine if there are any modifications necessary to ensure improved compliance [6, 8]

**Table 2:** Examples of African countries that have enacted legislation to control the use of plastic bags [2, 5, 6, 43].

Country	Year
Benin	2017
Botswana	2007 and 2017 (ban reinstated)
Burkina Faso	2014
Cameroon	2014
Cape Verde	2016
Chad	2005
Cote d'Ivoire	2014
Djibouti	2016
Egypt	2017
Eritrea	2005
Ethiopia	2016
Gabon	2010
Gambia	2015
Ghana	2015
Guinea-bissau	2016
Kenya	2007, 2011 and 2017 (ban reinstated)
Madagascar	2015
Malawi	2015
Mali	2013
Mauritania	2013
Mauritius	2016
Morocco	2015
Mozambique	2016
Niger	2014
Nigeria	2014
Republic of the Congo	2012
Rwanda	2004 and 2008 (ban reinstated)
Senegal	2015
Somalia	2005 and 2015 (ban reinstated)
South Africa	2004 (levy)
Tanzania	2006
Togo	2014
Tunisia	2017
Uganda	2007
Zimbabwe	2010

## Data Availability

The data are available from the corresponding author on reasonable request.

## References

[1] Geyer R., Jenna J. R., Law K. L. (2017). Production, use, and fate of all plastics ever made. *Science Advances*.

[2] Plastics Europe (2017). Breaking bag habits. https://ec.europa.eu/environment/efe/news/breaking-bag-habits-2017-11-24_en.

[3] Mugisha J., Diiro G. (2015). Households’ responsiveness to government ban on polythene carrier bags in Uganda. *Journal of Agriculture and Environmental Sciences*.

[4] Jambeck J., Diiro G., Hardesty B. D., Brooks A. L. (2018). Challenges and emerging solutions to the land-based plastic waste issue in Africa. *Marine Policy*.

[5] Behuria P. (2019). The comparative political economy of plastic bag bans in East Africa: why implementation has varied in Rwanda, Kenya and Uganda. *GDI Working Paper 2019-037*.

[6] Herbez T., Barlow C. Y., Finkbeiner M. (2020). Sustainability of single–use plastic bans. *Sustainability*.

[7] Plastic Oceans (2020). Plastic bags help the environment really?. https://plasticoceans.org/plastic-bags-help-the-environment-realy/.

[8] Chasse C. (2018). Evaluation of legal strategies for the reduction of plastic bag consumption.

[9] Danielsson M. (2017). The plastic bag ban in Rwanda: local procedures and successful outcomes.

[10] Owusu-Sekyere E., Osumanu I. K., Abdul-Kadri Y. (2013). An analysis of the plastic waste collection and wealth linkages in Ghana. *International Journal of Current Research*.

[11] Chitombe J. W. (2014). The plastic bag “ban” controversy in Zimbabwe: an analysis of policy issues and local responses. *International Journal of Development and Sustainability*.

[12] Dikgang J., Leiman A., Visser M. (2010). Analysis of the plastic-bag levy in South Africa. http://www.econrsa.org/papers/p_papers/pp18.pdf.

[13] Global Citizen (2019). Kenya bans single use plastics from beaches and parks. https://wwwglobalcitizen.org/en/content/single-use-plastics-banned-kenya-protected-areas.

[14] McLellan H. Banning the plastic shopping bag in South Africa–an idea whose time has come.

[15] Ramaswamy V., Sharma H. R. (2011). Plastic bags–threat to environment and cattle health: a retrospective study from gondar city of Ethiopia. *The IIOAB Journal*.

[16] Plastic Pollution Coalition (2017). How countries in Africa are wining the fight against plastic Bag. http://www.plasticpollutioncoalition.org.

[17] Gambrah P. (2013). Plastic bags waste management using the knapsack model, case study; trashy bags, accra. *International Journal of Scientific & Engineering Research*.

[18] Vince J., Hardesty B. D. (2017). Plastic pollution challenges in marine and coastal environments: from local to global governance. *Restoration Ecology*.

[19] Matsuguma Y., Takada H., Kumata H. (2017). Microplastics in sediment cores from Asia and Africa as indicators of temporal trends in plastic pollution. *Archives of Environmental Contamination and Toxicology*.

[20] Cancer Council (2018). *Cancer Myth: Plastic Food and Drink Packaging*.

[21] Barra R., Leonard S. A. (2018). *Plastics and the Circular Economy*.

[22] Ball A., Craig R. (2009). *Using Neo-Institutionalism to Advance Social and Environmental Accounting*.

[23] Brammer S., Jackson G., Matten D. (2012). Corporate Social Responsibility and institutional theory: new perspectives on private governance. *Socio-Economic Review*.

[24] Delbridge R., Edwards T. (2013). Inhabiting institutions: critical realist refinements to understanding institutional complexity and change. *Organization Studies*.

[25] Tolbert P. S. (1996). *The Institutionalization of Institutional Theory*.

[26] Knoblauch D., Mederake L., Stein U. (2018). Developing countries in the lead–what drives the diffusion of plastic bag policies?. *Sustainability*.

[27] Poortinga W., Whitmarsh L., Suffolk C. (2016). The english plastic bag charge: changes in attitude and behavior. http://orca.cf.ac.uk/94652/1/Cardiff-University-Plastic-Bag-Report-A4//28final/proof/.pdf.

[28] Kirsten E. (2019). Africa leads world in plastic bag ban. https://getaway.co.za.

[29] Martinho G., Balaia N., Pires A. (2017). The Portuguese plastic carrier bag tax: the effects on consumers’ behavior. *Waste Management*.

[30] Poortinga W., Whitmarsh L., Suffolk C. (2013). The introduction of a single-use carrier bag charge in Wales: attitude change and behavioural spillover effects. *Journal of Environmental Psychology*.

[31] Higgins J. P. T., Greens S. (2011). Cochrane handbook for systematic reviews of interventions. http://www.handbook.cochrane.org.

[32] Anfara V. A., Mertz N. T. (2006). *Theoretical Frameworks in Qualitative Research*.

[33] Collins C. S., Stockton C. M. (2018). The central role of theory in qualitative research. *International Journal of Qualitative Methods*.

[34] Berthod O. (2018). Institutional theory of organiza. *Global Encyclopedia of Public Administration, Public Policy, and Governance*.

[35] Thoenig J. (2011). *Institutional Theories and Public Institutions: New Agendas and Appropriateness*.

[36] Palthe J. (2014). Regulative, normative, and cognitive elements of organizations: implications for managing change. *Science and Education Press*.

[37] Hoffman A. J., Jennings P. D. (2015). Institutional theory and the natural environment: research in (and on) the anthropocene. *Organization & Environment*.

[38] Aten K., Howard-Grenville J. (2012). Encouraging trade at the boundary of organizational culture and institutional theory. *Journal of Management Inquiry*.

[39] Stewart R. (2015). A theory of change for capacity building for the use of research evidence by decision makers in Southern Africa. *Evidence & Policy: A Journal of Research, Debate and Practice, Policy Press*.

[40] Fakoya M. B. (2014). Institutional challenges to municipal waste management service delivery in South Africa. *Journal of Human Ecology*.

[41] Mungure J. M. (2008). *Governance and Community Participation in Municipal Solid Waste Management, Case of Arusha and Dar Es Salaam Tanzania, Department of Development and Planning*.

[42] Patton C., Sawicki D. (1993). *Basic Methods of Policy Analysis and Planning*.

[43] Green Africa (2020). 34 plastic bans in Africa: reality check. http://www.greenpeace.org/africa/en/blogs/11156/34-plastic-bans-in-africa/.

[44] McAllister J. (2015). *Factors Influencing Solid Waste Management in the Developing World, A Plan B Report Submitted in Partial Fulfilment of the Requirements for the Degree of Master of Science in Geography*.

[45] United Nations (2018). *Africa is on the Right Path to Eradicate Plastics*.

[46] Business Day (2018). *Bin the Bag: Business Can Lead the Offensive Against Plastic Waste*.

[47] Afrik21 (2019). *Plastic Waste: An Overview of Repressive Legislation in African Countries (1/2)*.

[48] Mogomotsi P. K. (2019). *Plastic Bag Usage in a Taxed Environment: Investigation on the Deterrent Nature of Plastic Levy in Maun, Botswana*.

[49] All Africa Com (2018). Rwanda against single-use plastics pending cabinet approval. https://allafrica.comstories.

[50] Kohls R. (2011). The plastic bag debate–lessons from rwanda, the dominion–news from the grassroots. http://www.dominionpaper.ca/articles/4010.

[51] Reuse This Bag.com (2020). Understanding plastic bag bans around the world. https://www.reusethisbag.com/articles/plastic-bag-bans-worldwide.

[52] The East African News (2017). Finally, Kenya effects ban on plastic bags. http://www.theeastafrican.co.ke/business/kenya-effects-ban-on-plastic-bags-/2560-4086512-view-asAM.

[53] Kurian J. (2013). Stakeholder participation for sustainable waste management. http://www.hia21.eu.dwnld/20131229-stakeholder-participation-for-sustainable-waste-management.

[54] Muthoni P. (2014). *Stakeholder and Solid Waste*.

[55] Madigele P. K. (2017). Consumer willingness to pay for plastic bags levy and willingness to accept eco-friendly alternatives in Botswana. *Chinese Journal of Population Resources and Environment*.

[56] Southern Times Africa (2018). Row over botswana ban on use and importation of plastic bags. https://southerntimesafrica.com/news.

[57] Mangizvo R. V. (2012). The incidence of plastic waste and their effects in alice, South Africa. *Online Journal of Social Sciences*.

[58] Business Tech (2019). Some of South Africa’s biggest shopping malls are banning plastic bags. https://businesstech.co.za/news.

[59] EMA (2019). Prohibition of plastics online. https://www.ema.co.zw/index.php.

[60] Scientific Action and Advocacy Network (2019). Effectiveness of plastic regulation around the world. http://scanna.net.

[61] He H. (2012). Effects of environmental policy on consumption: lessons from the Chinese plastic bag regulation. *Environment and Development Economics*.

